# Autocrine IL-10 activation of the STAT3 pathway is required for pathological macrophage differentiation in polycystic kidney disease

**DOI:** 10.1242/dmm.024745

**Published:** 2016-09-01

**Authors:** Jacqueline D. Peda, Sally M. Salah, Darren P. Wallace, Patrick E. Fields, Connor J. Grantham, Timothy A. Fields, Katherine I. Swenson-Fields

**Affiliations:** 1 Department of Pathology and Laboratory Medicine, University of Kansas Medical Center, Kansas City, KS 66160, USA; 2 Kidney Institute, University of Kansas Medical Center, Kansas City, KS 66160, USA; 3 Department of Anatomy and Cell Biology, University of Kansas Medical Center, Kansas City, KS 66160, USA; 4 Department of Internal Medicine, University of Kansas Medical Center, Kansas City, KS 66160, USA; 5 Department of Molecular and Integrative Physiology, University of Kansas Medical Center, Kansas City, KS 66160, USA

**Keywords:** PKD, Macrophages, Cyst, Proliferation, IL-10, STAT3

## Abstract

Polycystic kidney disease (PKD) is characterized by slow expansion of fluid-filled cysts derived from tubules within the kidney. Cystic expansion results in injury to surrounding parenchyma and leads to inflammation, scarring and ultimately loss of renal function. Macrophages are a key element in this process, promoting cyst epithelial cell proliferation, cyst expansion and disease progression. Previously, we have shown that the microenvironment established by cystic epithelial cells can ‘program’ macrophages, inducing M2-like macrophage polarization that is characterized by expression of markers that include *Arg1* and *Il10*. Here, we functionally characterize these macrophages, demonstrating that their differentiation enhances their ability to promote cyst cell proliferation. This observation indicates a model of reciprocal pathological interactions between cysts and the innate immune system: cyst epithelial cells promote macrophage polarization to a phenotype that, in turn, is especially efficient in promoting cyst cell proliferation and cyst growth. To better understand the genesis of this macrophage phenotype, we examined the role of IL-10, a regulatory cytokine shown to be important for macrophage-stimulated tissue repair in other settings. Herein, we show that the acquisition of the pathological macrophage phenotype requires IL-10 secretion by the macrophages. Further, we demonstrate a requirement for IL-10-dependent autocrine activation of the STAT3 pathway. These data suggest that the IL-10 pathway in macrophages plays an essential role in the pathological relationship between cysts and the innate immune system in PKD, and thus could be a potential therapeutic target.

## Abstract (precis)

**Summary:** Macrophages in polycystic kidney disease are induced by cyst epithelial cell factors to perform pathological pro-proliferative functions through stimulation of an autocrine IL-10–STAT3 pathway.

## INTRODUCTION

Polycystic kidney disease (PKD) is one of the most common potentially fatal genetic disorders. The most common form (autosomal dominant; ADPKD) affects up to ∼1 in 500 in the USA and is the fourth leading cause of adult end-stage kidney disease worldwide (Grantham, 2008; Torres et al., 2007). ADPKD is caused by inherited mutations in one of two genes – *PKD1* (∼85% of cases) or *PKD2* (∼15% of cases) – and is characterized primarily by numerous renal cysts, which enlarge continuously over the individual's lifetime. Loss-of-function mutations in these genes result in renal tubular epithelial cells that display aberrant proliferative and secretory properties, and form these characteristic fluid-filled cysts. Currently, no Food and Drug Administration (FDA)-approved specific therapies are available for PKD. Because the clinical course for PKD is generally quite slow, targeting factors that promote progression can potentially make a substantial clinical impact, especially if implemented early in the disease.

The pathway to renal failure in PKD is initiated by expanding cysts in kidneys, which continuously compress and distort the surrounding functioning parenchyma, resulting in obstruction, injury, atrophy and massive fibrosis. Consequently, the kidneys of PKD individuals are in a continuous state of chronic injury owing both to expanding cysts and the accompanying fibrosis, which ultimately results in renal failure (Grantham et al., 2011). Not surprisingly, a chronic inflammatory environment is present in cystic PKD kidneys, as evidenced by the large numbers of interstitial macrophages that we and others have shown to be present within cystic kidneys of both humans and rodents (Karihaloo et al., 2011; Prasad et al., 2009; Swenson-Fields et al., 2013).

A large majority of the macrophages in PKD kidneys of both human and mouse origin share phenotypic properties with M2 macrophages (i.e. those that arise *in vitro* from exposure to IL-4 and/or IL-13) (Karihaloo et al., 2011; Lee et al., 2011; Swenson-Fields et al., 2013). Following acute renal injury, similar ‘M2-like’ macrophages are known to accumulate in the kidney in large numbers. These cells originate from both renal macrophage proliferation and bone-marrow-derived monocytes, which are prompted to differentiate and acquire an M2-like phenotype in response to local renal cues (Duffield, 2011; Zhang et al., 2012). These M2-like macrophages are known to promote repair, proliferation and regeneration of damaged tissues. Following repair, macrophage numbers decline to those found in the pre-injured state. However, in the case of chronic injury, the M2-like macrophages persist, where they promote fibrosis (Anders and Ryu, 2011; Huen and Cantley, 2015; Ricardo et al., 2008).

Using multiple mouse models of PKD, we and others have demonstrated that the presence of these macrophages in cystic kidneys promotes tubule cell proliferation, cyst expansion and disease progression (Karihaloo et al., 2011; Swenson-Fields et al., 2013). We have postulated that these macrophages in PKD kidneys could have arisen in response to the ongoing renal injury in a similar manner to those that arise following acute renal injury (Swenson-Fields et al., 2013). However, rather than being reparative, the tubule cell proliferation that occurs in response to their presence is maladaptive and pathological, promoting cyst expansion.

The molecular cues and cellular pathways that promote the development of the macrophages in PKD kidneys are incompletely understood. Evidence from a recent study has demonstrated that tubular epithelial cells secrete factors that promote the M2-like macrophage phenotype following acute kidney injury. In these studies, conditioned media from primary tubule epithelial cells were shown to program macrophages to assume an mRNA expression profile that mimicked the M2-like profile found *in vivo* following ischemia-reperfusion (I-R) injury (Huen et al., 2015). However, direct effects of this programming on macrophage effector functions, including potential effects on macrophage pro-proliferative activity (i.e. the ability of macrophages to induce the proliferation of other cells), were not examined. Similarly, we have found that primary ADPKD cells and their soluble factors can program macrophages to acquire a transcriptional profile *in vitro* that is M2-like, and thus might provide a source of the differentiation cues that promote the appearance of the M2-like macrophages in cystic kidneys *in vivo* (Swenson-Fields et al., 2013). Moreover, using both direct and Transwell-insert co-cultures of macrophages with primary ADPKD cyst cells, we have shown not only that the macrophages acquired an M2-like gene expression profile but also that the presence of macrophages in these co-cultures promoted proliferation of the tubule epithelial cells. One possibility to explain these results is that the programming of macrophages by ADPKD cells alters not only the marker phenotype but also the functional properties of these cells, converting them to those that produce pro-proliferative factors. However, because we were unable to replicate the pro-proliferative activity using the culture conditions employed in those experiments, the cellular origin of these factors (macrophages or tubule cells in the presence of macrophages) is unresolved (Swenson-Fields et al., 2013).

In this study, using a refined *in vitro* culture system to study the interaction between cyst cells and macrophages, we demonstrate that soluble factors produced by primary ADPKD cyst epithelial cells program macrophages to become cells with enhanced pro-proliferative capacity, producing soluble macrophage factors that boost cell division of tubule epithelial cells, when compared with factors from unprogrammed resting macrophages. Moreover, we show that autocrine IL-10, which is induced in macrophages during this programming, and the autocrine IL-10–STAT3 pathway are required for acquisition of this pro-proliferative phenotype. Further evidence is provided to suggest that this pathway is operational in human ADPKD kidneys *in vivo*. These results indicate that the IL-10–STAT3 pathway in macrophages could be a good target for the development of effective therapies to block macrophage-promoted cyst expansion and accordingly slow ADPKD disease progression.

## RESULTS

### Primary cyst epithelial cells program macrophages to acquire a pro-proliferative pathological phenotype

**Fig. 1.**
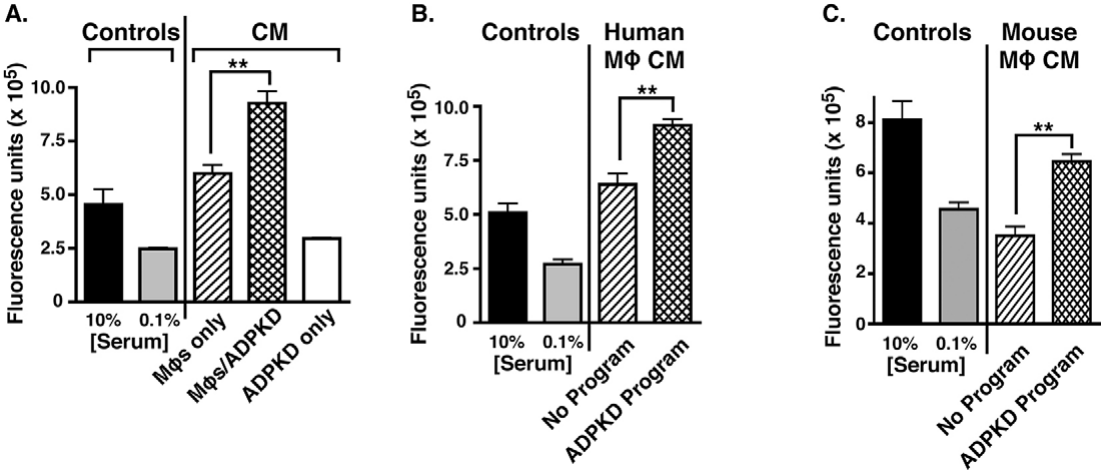
**Both co-culture of macrophages with ADPKD cyst cells and macrophages programmed through exposure to ADPKD-CM produce secreted factors that have enhanced pro-proliferative activity.** (A) PKD-A cells were incubated with CM obtained from THP-1 macrophages that had been cultured alone (MΦs only) or from macrophages that had been co-cultured with ADPKD cyst cells (MΦ/ADPKD), or ADPKD cyst cells that had been cultured alone (ADPKD only) for 72 h, and proliferation of PKD-A cells was measured using CyQUANT^®^ GR dye as described in Materials and Methods. For comparison, baseline proliferation was assessed in basal medium containing 0.1% FBS, and stimulated proliferation was assessed with 10% FBS (two bars on the left). Representative results from an experiment are shown. Similar results were obtained in at least five independent experiments using cyst cells in co-culture from different ADPKD individuals. (B) PKD-A cells were incubated with CM obtained from THP-1 macrophages that had been cultured alone (No Program) or from those programmed by incubation with ADPKD cyst cells as described in Materials and Methods. After 72 h, PKD-A cells were collected and assayed for proliferation as described in A. Similar results were observed in at least five independent experiments using CM generated with cyst cells from different ADPKD individuals. (C) PKD-A cells were incubated with CM obtained from RAW macrophages that had been cultured alone (No Program) or from those programmed by incubation with ADPKD-CM, and proliferation was measured as described in A. Similar results were observed in at least five independent experiments using CM generated with cyst cells from different ADPKD individuals. Data in A-C are presented as mean±s.e.m. of three technical replicates for each experiment. Means were compared using *t*-test; **P<0.01.

To examine the potential effects of cyst cells on the macrophage functional phenotype during PKD, we developed an *in vitro* system wherein macrophages are first exposed to cyst cells (‘programmed’) and the macrophage-secreted factors subsequently produced are assayed for their ability to influence cyst cell proliferation. Because serum contains factors that are likely to influence macrophage phenotype, serum was largely omitted from the culture conditions (see Materials and Methods). Using these serum-free conditions, we first tested whether co-cultures of macrophages and ADPKD cyst cells could produce transferable soluble factors that would promote enhanced proliferation of cyst cells. For these experiments, human macrophages that had been differentiated from a monocyte-like cell line (THP-1) were first co-cultured with ADPKD cells, to allow programming, or were cultured alone to remain unprogrammed. Following a change into basal medium, these co-cultures were further incubated for an additional day to allow for secretion of cellular factors. The now conditioned media (CM) from these cultures were collected and incubated with ADPKD cells in a proliferation assay (Fig. 1A). Little proliferation was conferred by the CM of ADPKD cells that had been cultured alone, whereas the CM from both co-cultures containing macrophages and macrophages that had been cultured alone were stimulatory in this assay (Fig. 1A). Furthermore, the CM from co-cultures that contained the now programmed macrophages provided a significantly elevated level of proliferation relative to unprogrammed macrophage CM. The enhanced proliferative effects of these programmed macrophage co-cultures were not specific for ADPKD cells; similar results were observed in proliferation assays from co-cultures with tubule epithelial cells that had been derived from non-cystic human kidneys (NHK; data not shown). This was not surprising because we have previously shown that NHK cells also promote M2-like differentiation (Swenson-Fields et al., 2013). These results indicate that macrophage–ADPKD co-cultures produce soluble factors that have enhanced pro-proliferative activity when compared with the secreted products from either cell type alone.

To determine whether macrophages are responsible for the enhanced pro-proliferative activity in the co-culture CM, macrophages were programmed with serum-free ADPKD-cyst-cell-conditioned media (ADPKD-CM), rather than direct co-culture. Following this programming, the ADPKD-CM was washed out and replaced with basal medium for 24 h. The CM from macrophages that had been programmed in this manner were then collected and tested for proliferative effects on ADPKD cells (Fig. 1B). Although both unprogrammed and programmed macrophage CM promoted proliferation, the programmed macrophage CM showed enhanced activity. In addition, similar programming of mouse macrophages (RAW 264.7 cells, a macrophage-like cell line; hereafter referred to as RAW cells) with ADPKD-CM resulted in a similar enhanced pro-proliferative phenotype (Fig. 1C). These results indicate that ADPKD cells (or NHK cells) secrete factor(s) that program macrophages to acquire an enhanced pro-proliferative phenotype, one that would be predicted to be pathological in PKD.

### Autocrine IL-10 is required but is not sufficient for pathological macrophage differentiation

In a previous study, we have shown that macrophages programmed through exposure to primary ADPKD cells or NHK cells, or to CM from these cell types, have enhanced transcription and protein expression of the cytokine IL-10 in comparison to unprogrammed macrophages (Swenson-Fields et al., 2013). Although IL-10 is best known as an immunomodulatory cytokine with anti-inflammatory properties, it has also been implicated in altering the macrophage phenotype to one that promotes proliferation in some cellular contexts (Apte et al., 2006; Dace et al., 2008; Deng et al., 2012; Jung et al., 2012a; Kelly et al., 2007; Nakamura et al., 2015). Thus, it was of interest to determine the contribution of IL-10 to the acquisition of an enhanced pro-proliferative macrophage phenotype elicited through programming with ADPKD cells or ADPKD-cell-CM.

**Fig. 2.**
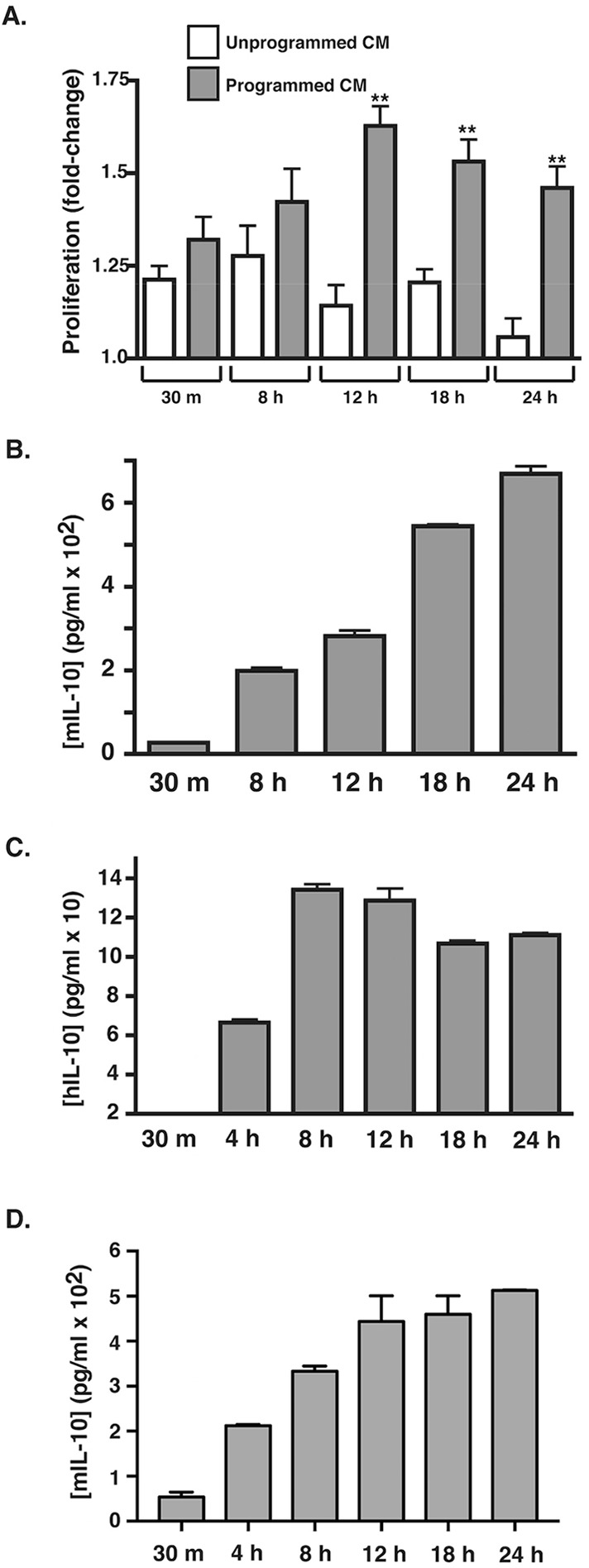
**Timing of induction of the pathological macrophage phenotype by ADPKD programming coincides with induction of IL-10 secretion.** (A) RAW macrophages were incubated in duplicate with medium or ADPKD-CM for the indicated times. Media were collected and stored at 4°C, and cells were then washed and incubated with basal medium for 24 h to produce programmed or unprogrammed CM, which were then assayed for the ability to stimulate proliferation of PKD-A cells, as described in Materials and Methods. Data are presented as the fold change relative to the baseline proliferation determined from PKD-A cells grown in 0.1% FBS in parallel. Similar results were observed in three independent experiments using CM generated with cyst cells from different ADPKD individuals. (B) RAW-macrophage-produced IL-10 levels were measured in the collected ADPKD-CM that was used for the programming described in A with the use of an ELISA kit specific for mouse IL-10. Similar results were observed in three independent experiments using CM generated with cyst cells from different ADPKD individuals. (C) THP-1 macrophages were incubated with ADPKD-CM for the times indicated, and IL-10 secretion was measured by using ELISA. Human IL-10 was not detectable in either the ADPKD-CM used for programming or in medium from unprogrammed macrophages (data not shown). (D) Mouse BMDMs were programmed in duplicate for the time indicated with primary NHK-conditioned medium. Media were collected, and IL-10 concentration was measured by using ELISA. hIL-10, human IL-10; mIL-10, mouse IL-10. Data are mean±s.e.m. **P<0.01 (two-tailed *t*-test).

First, the timing for both macrophage programming and the stimulation of IL-10 production was determined and compared. For these studies, we first programmed RAW macrophages through incubation with ADPKD-CM or with control medium, after which: (1) the medium was collected over time for measurement of mouse IL-10 protein concentrations; and in parallel cultures, (2) the medium was washed out, and macrophages were incubated for a further 24 h in basal medium to produce CM that were assessed for pro-proliferative activity. In these experiments, enhanced macrophage-produced pro-proliferative activity was not manifested until ∼12 h of programming had taken place (Fig. 2A). During the programming, high levels (∼200 pg/ml) of mouse IL-10 were detected at 8 h, and these levels continued to increase throughout the programming period (Fig. 2B). High levels of IL-10 (∼70-130 pg/ml) were also produced by both THP-1 macrophages (Fig. 2C) and primary mouse bone-marrow-derived macrophages (BMDMs; Fig. 2D) that had been programmed in a similar manner. IL-10 was not detected (<30 pg/ml) in unprogrammed human or mouse macrophage CM (not shown). IL-10 was also undetectable or low (<40 pg/ml) in the 24-h CM collected from programmed macrophages that was used to assay pro-proliferative activity (data not shown), indicating that the production of IL-10 during programming is transient and is unlikely to contribute directly to the pro-proliferative activity generated. In fact, neither mouse nor human IL-10 was capable of stimulating ADPKD cell proliferation (see below). Collectively, these results demonstrate that measurable levels of IL-10 are produced by macrophages during programming before the development of the enhanced pro-proliferative phenotype. Thus, a contribution of IL-10 to the acquisition of this phenotype is possible.

**Fig. 3.**
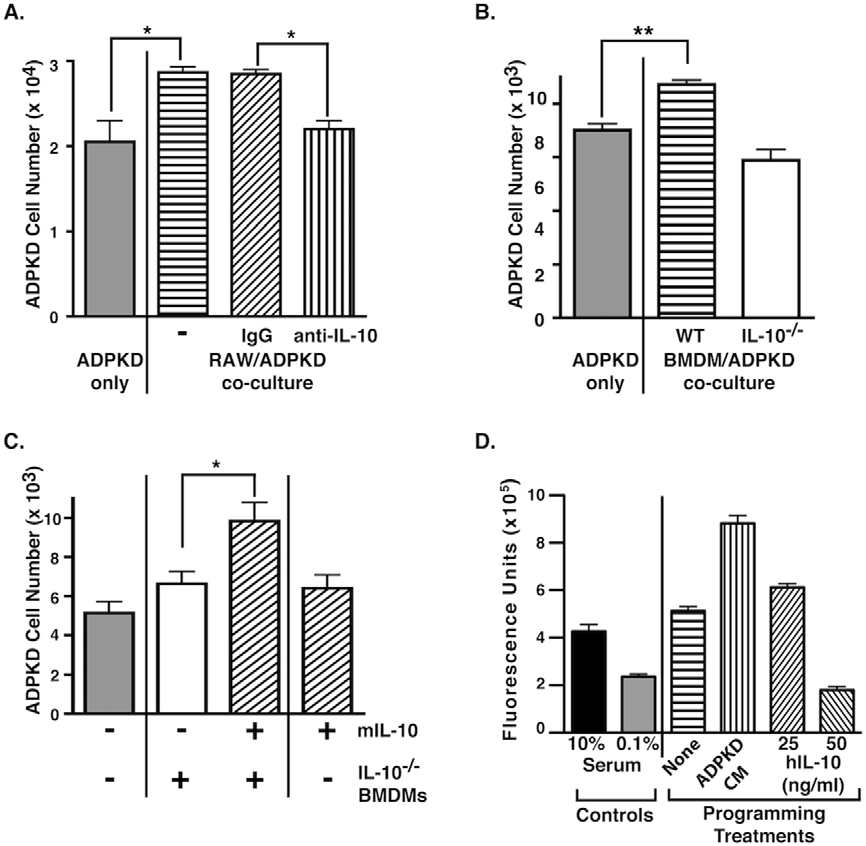
**Macrophage-produced IL-10 is required but not sufficient for macrophage-stimulated proliferation of ADPKD cells.** (A) ADPKD cyst cells were either cultured in 0.1% FBS (left bar) or directly co-cultured with RAW macrophages in the presence of either a mouse-specific IL-10-neutralizing antibody (2 µg/ml) or a non-immune IgG control antibody (2 µg/ml). After 72 h, cells were trypsinized and fixed, and ADPKD cell proliferation determined by direct counting. This experiment was repeated using ADPKD cells from more than three different kidneys with similar results. (B) ADPKD cyst cells were either cultured in 0.1% FBS (left bar) or indirectly co-cultured (in Transwell inserts) with identical numbers of BMDMs that had been isolated from either wild-type (WT) or IL-10-deficient (IL-10^−/−^) mice. After 72 h, cells were trypsinized, fixed, and ADPKD cell proliferation was determined by direct counting. This experiment was repeated using ADPKD cells from more than three different kidneys and BMDMs from more than three different WT and IL-10^−/−^ mice with similar results. (C) ADPKD cyst cells were either cultured in 0.1% FBS (left bar) or indirectly co-cultured (in Transwell inserts) with BMDMs that had been isolated from IL-10^−/−^ mice in the presence or absence (middle) of added recombinant mouse IL-10 (20 ng/ml) for 72 h. ADPKD cells were also cultured with 0.1% FBS plus mouse IL-10 alone (right). Proliferation was assayed by direct counting. This experiment was repeated using ADPKD cells from three different kidneys with similar results. (D) THP-1 macrophages were cultured either alone (None), with ADPKD-CM, or the indicated concentrations of IL-10 for 72 h. Cells were then washed and incubated with basal medium for 24 h to produce CM, which was incubated with ADPKD cyst cells for 72 h. Proliferation was measured using CyQUANT^®^ GR dye. In parallel, cells were incubated with 0.1% and 10% FBS, to measure baseline and stimulated proliferation, respectively (left bars). This experiment was repeated using ADPKD cells from four different kidneys with similar results. hIL-10, human IL-10; mIL-10, mouse IL-10. All data are represent mean±s.e.m. Means were compared using *t*-test; *P<0.05 and **P<0.01.

To test directly whether the IL-10 produced during macrophage programming contributes to the acquisition of the pro-proliferative phenotype, the proliferation of ADPKD cells was measured following direct co-culture with RAW macrophages in the presence of a mouse-specific IL-10-neutralizing antibody (Fig. 3A). The presence of the neutralizing antibody in the co-culture attenuated ADPKD cell proliferation to levels similar to those measured for cells that had been incubated in low-serum conditions alone, whereas control IgG had no effect (Fig. 3A). These results suggest that IL-10 produced by macrophages during programming is required for the stimulation of the ADPKD cell proliferation that is induced by these immune cells when in co-culture.

To confirm this conclusion, proliferation of ADPKD cells was also measured following their co-culture with BMDMs isolated from wild-type mice or mice that were genetically deficient for IL-10 (Fig. 3B). Similar to the effects of RAW macrophages, wild-type BMDMs significantly stimulated ADPKD cell proliferation in these co-cultures. In contrast, equivalent numbers of IL-10-deficient BMDMs were unable to stimulate cyst cell proliferation (Fig. 3B). The addition of mouse IL-10 to co-cultures containing IL-10-deficient BMDMs rescued the pro-proliferative effect (Fig. 3C), whereas the addition of either mouse or human IL-10 alone to ADPKD cells did not stimulate proliferation (Fig. 3C; Fig. S1). Thus, these results demonstrate that the macrophage-secreted IL-10 is required during ADPKD-cell-mediated programming for the generation of the pro-proliferative activity produced by the macrophages but does not contribute directly to this activity.

To assess whether IL-10 might be sufficient for programming macrophages to a pro-proliferative phenotype, THP-1 macrophages were incubated with recombinant human IL-10, ADPKD-CM or medium alone. This was followed by wash-out and replacement with basal medium for 24 h prior to collection of CM, which were then tested for proliferative effects on ADPKD cells (Fig. 3D). Although ADPKD-CM-programmed macrophage CM demonstrated an enhanced proliferative activity as expected, the CM from IL-10-treated macrophages did not demonstrate proliferative activity greater than that seen with CM from control-medium-treated macrophages. Programming with high concentrations of IL-10 in fact blunted the pro-proliferative activity (Fig. 3D). Similar results were obtained using mouse IL-10 and RAW macrophages (data not shown). Thus, although macrophage-secreted IL-10 that is produced as a result of programming with ADPKD cells is required for the generation of pro-proliferative activity (Fig. 3A-C), it is not sufficient (Fig. 3D). In addition to IL-10, it is likely that other factors either produced by the macrophages themselves or present in the ADPKD CM are required to stimulate macrophages to acquire a pro-proliferative phenotype.

### IL-10-stimulated activation of STAT3 is required for pathological macrophage differentiation

IL-10 is known to signal through its receptor complex to stimulate tyrosine-residue phosphorylation and activation of STAT3 (Donnelly et al., 1999). To determine whether this signaling pathway is relevant to IL-10-dependent programming of macrophages, macrophages were incubated with ADPKD-CM and collected at successive time points. The relative levels of activated STAT3 were determined by immunoblot analysis using a phosphorylation (phospho)-specific STAT3 antibody.

**Fig. 4.**
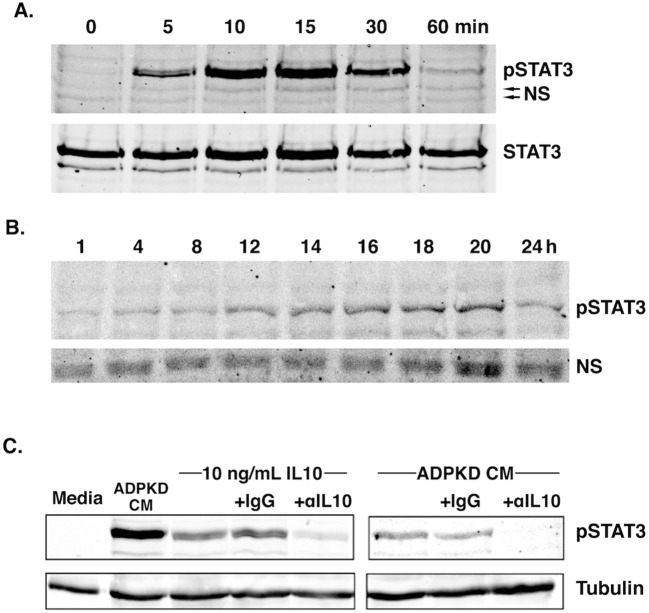
**Programming of macrophages with ADPKD-CM promotes IL-10-dependent STAT3 activation.** (A,B) RAW macrophages were incubated with ADPKD-CM for the time indicated before lysis and immunoblot analyses using anti-phospho-STAT3 (pSTAT3) and anti-STAT3 (STAT3) antibodies. (A) Immunoblot of cell samples collected at 0-60 min. (B) Immunoblot of cell samples collected at 1-24 h. NS, non-specific bands, which serve as a control for protein loading. Similar results were observed in three independent experiments using CM generated with cyst cells from different ADPKD individuals. (C) RAW macrophages were incubated for 18 h with either mouse IL-10 (10 ng/ml), ADPKD-CM or medium alone, as indicated, in the presence of an anti-IL-10 neutralizing antibody (αIL10) or equal concentrations of a control antibody (IgG) before cell lysis. Total cell lysates were subjected to immunoblot analyses using an anti-phospho-STAT3 antibody, as well as anti-tubulin antibodies for a control. Similar results were observed in three independent experiments using CM generated with cyst cells from different ADPKD individuals.

Treatment of RAW macrophages with ADPKD-CM led to an early but transient phosphorylation of STAT3, which was detectable after 5 min and peaked after 15 min, diminishing to near baseline levels by 1 h (Fig. 4A). Upon longer incubation, there was an increase in STAT3 phosphorylation starting ∼8-12 h, which continued to rise, peaking at ∼18-20 h before declining at 24 h (Fig. 4B). Similar results showing an early and transient phosphorylation of STAT3, and a later peak phosphorylation, were obtained with THP-1 macrophages (Fig. S2). Providing further evidence of STAT3 activation in these cells was the observation that ADPKD-CM-mediated programming also significantly upregulated expression of *Socs3* (Fig. S3), an important downstream target of the IL-10–STAT3 pathway that is known to mediate many of the regulatory and inflammatory properties of IL-10 (Yin et al., 2015). The presence of STAT3 phosphorylation at later time periods (∼8-12 h) of programming in these macrophages roughly coincided with the timing of their IL-10 production (Fig. 2B-D), suggesting that this cytokine is the cause of the STAT3 pathway at these later time points.

To determine whether IL-10 produced by macrophages during ADPKD-CM-mediated programming contributes to the late activation of STAT3, experiments were conducted using the neutralizing anti-IL-10 antibody. The neutralizing antibody, but not control IgG, blocked STAT3 activation in RAW macrophages in response to treatment with recombinant IL-10, as expected (Fig. 4C, left panel). Similarly, the neutralizing antibody prevented activation of STAT3 by ADPKD-CM after 18 h, whereas control IgG, again, had no effect (Fig. 4C, right panel). These results indicate that IL-10 produced by macrophages during programming with ADPKD-CM is responsible for the late activation of the STAT3 pathway that occurs in these cells.

**Fig. 5.**
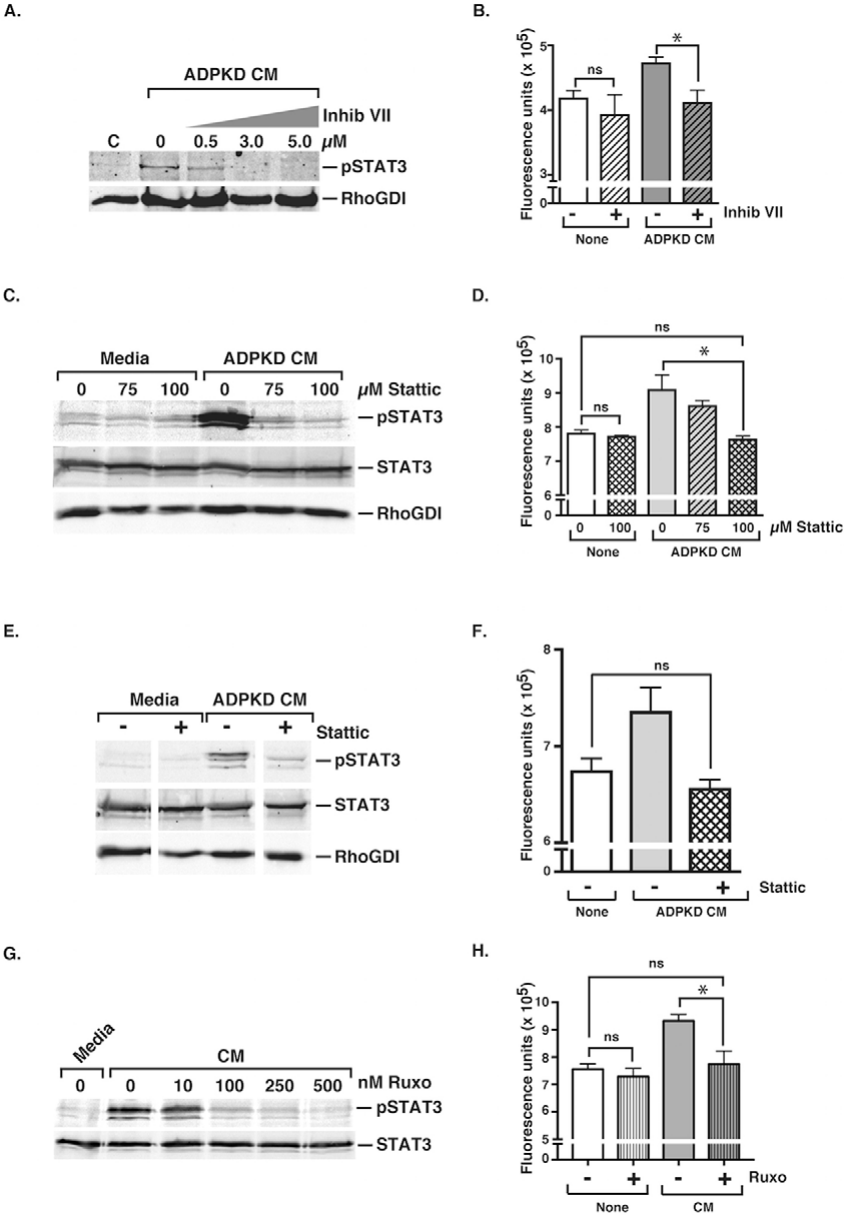
**Inhibition of macrophage STAT3 activation during ADPKD programming blunts development of the pathological pro-proliferative macrophage phenotype.** (A) STAT3 inhibitor VII (Inhib VII) blocks STAT3 activation in RAW macrophages during programming with ADPKD-CM in a dose-dependent manner. RAW macrophages were incubated in either medium (C) or ADPKD-CM for 18 h in the absence or presence of increasing concentrations of STAT3 inhibitor VII. Total cell lysates were subjected to immunoblot analyses using anti-phospho-STAT3 antibodies and anti-RhoGDI antibodies as a control. pSTAT3, phospho-STAT3. (B) STAT3 inhibitor VII inhibits development of the RAW macrophage pro-proliferative phenotype. Macrophages were incubated in either medium (None) or ADPKD-CM for 18 h in the absence or presence of STAT3 inhibitor VII (3 µM). Media were then removed, and cells were incubated for a further 24 h in basal medium, which was then collected and incubated with ADPKD cells for 72 h, and proliferation was measured with CyQUANT^®^ GR dye. Similar results were observed in three independent experiments using CM generated with cyst cells from different ADPKD individuals. (C) Stattic blocks STAT3 activation in THP-1 macrophages during programming with ADPKD-CM in a dose-dependent manner. THP-1 macrophages were incubated in either medium (C) or ADPKD-CM for 18 h in the absence or presence of increasing concentrations of Stattic before cell lysis. Total protein was subjected to immunoblot analyses using antibodies against the indicated proteins. (D) Stattic inhibits development of the THP-1 macrophage pro-proliferative phenotype. THP-1 macrophages were incubated in either medium (None) or ADPKD-CM for 18 h in the absence or presence of Stattic. Media were then removed, and cells were incubated for a further 24 h in basal medium, which was then collected and incubated with ADPKD cells for 72 h, and proliferation was measured with CyQUANT^®^ GR dye. Similar results were observed in three independent experiments using CM generated with cyst cells from different ADPKD individuals. (E) Addition of Stattic to THP-1 macrophage cultures after the first hour of programming blunts late activation of STAT3. THP-1 macrophages were incubated in either medium (C) or ADPKD-CM for 1 h prior to addition of Stattic (100 µM) or DMSO control, and were incubated further for 17 h. Cell lysates were subjected to immunoblot analyses using the indicated antibodies. (F) Stattic blockade of late STAT3 activation is sufficient to inhibit development of the THP-1 macrophage pro-proliferative phenotype. THP-1 macrophages were treated as described in E and incubated for a further 24 h in basal medium, which was then collected and tested for proliferative effects on ADPKD cells, as described in D. Similar results were observed in three independent experiments using CM generated with cyst cells from different ADPKD individuals. (G) Ruxolitinib (Ruxo) blocks STAT3 activation in THP-1 macrophages during programming with ADPKD-CM in a dose-dependent manner. THP-1 macrophages were incubated in either medium or ADPKD-CM for 18 h in the absence or presence of increasing concentrations of ruxolitinib before cell lysis. Total protein was subjected to immunoblot analyses against the indicated proteins. (H) Ruxolitinib inhibits development of the THP-1 macrophage pro-proliferative phenotype. THP-1 macrophages were incubated in either medium (None) or ADPKD-CM for 18 h in the absence or presence of ruxolitinib (500 nM). Media were then removed, and cells were incubated for a further 24 h in basal medium, which was then collected and incubated with ADPKD cells for 72 h, and proliferation was measured with CyQUANT^®^ GR dye. Data are means±s.e.m. *P<0.05; ns, not significant (two-tailed *t*-test).

A key question is whether the STAT3 pathway that is activated in macrophages during programming contributes to the generation of the pathological pro-proliferative phenotype. To address this question, selective inhibitors of STAT3 were utilized. Using RAW macrophages, the concentration of STAT3 inhibitor VII (Xu et al., 2008) required to block STAT3 activation in response to ADPKD-CM was first determined (Fig. 5A). Using this determined concentration (3 µM), macrophages were then incubated in the presence or absence of STAT3 inhibitor VII during programming with ADPKD-CM, and the effects on the pro-proliferative activity were measured (Fig. 5B). Although there was no significant effect of this drug on the proliferation-inducing activity of CM from unprogrammed macrophages, the STAT3 inhibitor blocked the induction of pro-proliferative activity caused by ADPKD-CM-mediated programming (Fig. 5B). A different STAT3 inhibitor, Stattic (Schust et al., 2006), blocked STAT3 activation induced by ADPKD-CM in THP-1 macrophages to a similar extent (Fig. 5C). Moreover, this inhibitor also blocked the proliferative activity of CM collected from macrophages that had been programmed with ADPKD-CM, without affecting the proliferation-inducing activity of CM collected from unprogrammed macrophages (Fig. 5D).

To determine whether the early (i.e. in the first hour; see Fig. 4A) or late (after 1 h; see Fig. 4B) activation of STAT3 during macrophage programming was important for the acquisition of a pro-proliferative macrophage phenotype, Stattic was incubated with THP-1 macrophages starting at 1 h after the addition of ADPKD-CM. The addition of Stattic at this time, again, blocked both the activation of STAT3 when measured at 18 h (Fig. 5E) and the induction of pro-proliferative activity (Fig. 5F). These results suggest that the early STAT3 activation is not sufficient and that the late STAT3 activation – i.e. that initiated by IL-10 – is required for the generation of the pro-proliferative activity of macrophages in response to programming with ADPKD-CM. Because activated STAT3 can potentially upregulate IL-10 production (Hedrich and Bream, 2010), it is possible that the early STAT3 peak (i.e. in the first hour; Fig. 4A) in programming affects the late peak at 18-20 h by regulating IL-10 production and the late STAT3 activation. Thus, the early activation of STAT3, although not sufficient, might contribute to the programming-induced conversion of macrophages into a pro-proliferative phenotype.

To further demonstrate the role of the autocrine IL-10–STAT3 pathway in pathological macrophage differentiation, we also examined the contribution of JAK1. This kinase is known to be activated by ligand-bound IL-10 receptor, which subsequently facilitates recruitment and activation of STAT3 (Murray, 2007). THP-1 macrophages were programmed with ADPKD-CM in the presence or absence of ruxolitinib, a potent inhibitor of JAK1 and JAK2 (Quintas-Cardama et al., 2010). As with the STAT3 inhibitors, the JAK inhibitor blocked both the macrophage STAT3 activation and pathological macrophage differentiation induced during ADPKD-CM-mediated programming (Fig. 5G-H).

### IL-10 is upregulated in ADPKD tissue and is present in cyst fluid

**Fig. 6.**
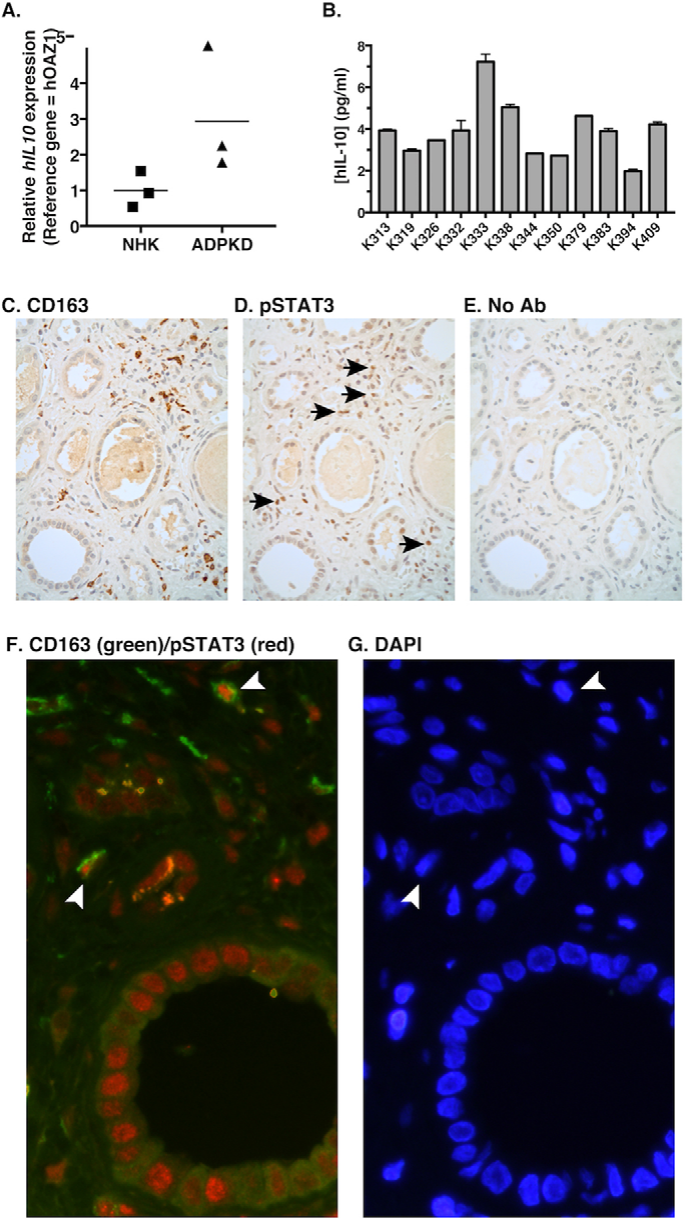
**Elevated *IL10* expression and STAT3 activation in ADPKD kidney macrophages.** (A) Elevated *IL10* expression in human ADPKD kidneys. RNA was isolated from NHKs and non-cystic regions of ADPKD kidneys (three of each). *IL10* expression was analyzed by performing qRT-PCR. The line indicates the mean relative value. (B) IL-10 is present in ADPKD cyst fluid. IL-10 in pooled cyst fluid collected from ADPKD kidneys from 12 different individuals was measured by using ELISA. Data are mean±s.e.m. (C-G) Phospho-STAT3 was present in macrophages of cystic ADPKD kidneys. Formalin-fixed, paraffin-embedded tissues from ADPKD kidneys were serially sectioned (C-E), and consecutive sections were stained by using immunohistochemistry with antibodies against the M2 macrophage marker CD163 (C), phospho-STAT3 (D) or secondary antibody only (E, No Ab) as a control. Arrows indicate phospho-STAT3-positive cells in macrophage-rich regions of interstitium. (F,G) Sections of ADPKD kidneys that had been fixed and prepared in a similar manner were co-stained with anti-CD163 (green) and anti-phospho-STAT3 (red) antibodies, followed by incubation with two distinct fluorescence-labeled secondary antibodies, each with the appropriate antibody specificity (F), and with DAPI (G). Arrowheads indicate co-staining cells. *n*=3 biological replicates. hIL-10, human IL-10.

To determine whether the IL-10 is relevant *in vivo* in human ADPKD, we used quantitative reverse-transcriptase PCR (qRT-PCR) to measure *IL10* mRNA levels in kidney tissues from ADPKD-affected individuals, as well as from NHK tissues. This analysis revealed increased *IL10* expression in all ADPKD kidney tissues analyzed when compared with NHK tissues, although statistical analysis did not show significance (*P*=0.118; Fig. 6A). Although we suspect that macrophages are the primary source of these transcripts, we were unable to confirm this through IL-10 immunohistochemistry analysis, which failed to show substantial signal over that of background. This was perhaps, in part, related to the fact that IL-10 is a secreted molecule. In any event, we also assessed IL-10 levels in cyst fluids. An enzyme-linked immunosorbent assay (ELISA) for IL-10 revealed measurable cytokine levels in all of the specimens, ranging from ∼2-7 pg/ml (Fig. 6B). Collectively, these data demonstrate that IL-10 is upregulated and expressed in human ADPKD kidneys.

### Activated STAT3 is present in macrophages of cystic ADPKD kidneys

To determine whether macrophage STAT3 activation is relevant in human ADPKD, sections of ADPKD cystic kidneys were stained using antibodies specific for macrophage markers and phosphorylated STAT3. To stain macrophages, we used an antibody specific for CD163, a marker of M2-like macrophages that we have previously shown reacts with ∼85-90% of the macrophages present in these kidneys (Swenson-Fields et al., 2013). Initially, individual antibodies (anti-CD163 or anti-phospho-STAT3) were used for immunohistochemical staining of adjacent serial sections of ADPKD cystic kidneys to look for interstitial phospho-STAT3-positive cells, specifically in macrophage-rich areas. In most macrophage-rich areas, nuclear phospho-STAT3-positive cells were detected, whereas few, if any, of these cells were detected in areas of interstitium devoid of macrophages (Fig. 6C,D). Phospho-STAT3-staining in the nucleus could also be detected within scattered cyst epithelial cells, as has been noted previously by several groups (Leonhard et al., 2011; Takakura et al., 2011; Talbot et al., 2011). Sections of ADPKD cystic kidneys were also prepared for immunofluorescence co-staining for CD163 and phospho-STAT3. In these preparations, occasional CD163-positive cells were identified (∼30-50/field collected from 5-7 random microscopy images taken with the 40× objective; not shown). Among these, rare cells (∼1.2/field) also showed phospho-STAT3 staining in the nucleus (Fig. 6F; Fig. S4). These represented ∼2.6% of the CD163-positive cells (Fig. S4). These results indicate that STAT3 activation is present in a subset of renal macrophages that are present in cystic kidneys *in vivo*.

## DISCUSSION

By assessing known markers of the macrophage phenotype, previous studies have suggested that renal NHK and ADPKD cyst epithelial cells program macrophages to become M2-like, similar to the phenotype found in renal macrophages in cystic ADPKD kidneys (Swenson-Fields et al., 2013). This programming scenario is similar to that demonstrated for renal macrophages following I-R injury, wherein tubular epithelial cells instruct macrophages to assume an mRNA expression profile largely corresponding to the M2-like profile found for these cells *in vivo* (Huen et al., 2015). Depletion of these M2-like macrophages following I-R injury worsens subsequent tubule cell proliferation and repair (Lee et al., 2011), suggesting that these cells have a functional pro-proliferative phenotype. However, experimental evidence demonstrating direct effects of tubular epithelial cell instruction on the effector functions of macrophages, and particularly pro-proliferative functions, is lacking.

Here, we demonstrate that the programming of macrophages by primary ADPKD cells (or NHK cells) alters macrophage function by inducing the acquisition of an enhanced pro-proliferative phenotype. These cells might be similar to those M2-like macrophages that develop within the kidney in response to I-R injury and that are known to promote tissue repair (Lee et al., 2011). In PKD cystic kidneys, pro-proliferative macrophages are likely to be maladaptive and pathological, promoting cyst epithelial cell proliferation and cyst expansion rather than promoting repair.

This study has also demonstrated a key role for IL-10 in driving the conversion of macrophages to an enhanced pro-proliferative phenotype during programming. Macrophage IL-10 produced in response to ADPKD-CM-mediated programming (Swenson-Fields et al., 2013) that acts in an autocrine fashion was required for acquisition of the pro-proliferative pathological phenotype. Although IL-10 is best known as an anti-inflammatory and immunosuppressive cytokine that affects both macrophages and other immune cells (Moore et al., 2001), this cytokine has also has been shown to promote macrophage-stimulated proliferation in a variety of settings. In a recent study, macrophages overexpressing IL-10 were adoptively transferred into rats following I-R injury and found to stimulate not only anti-inflammatory effects in the kidney but also renal epithelial proliferation and regeneration (Jung et al., 2012a).

IL-10-dependent macrophage signaling has also been demonstrated to be an important contributor to proliferation and disease in other organs. For example, IL-10-dependent macrophage signaling has been found to be important in pathological vascular proliferation in the eye (Apte et al., 2006; Kelly et al., 2007; Nakamura et al., 2015). In these studies, investigators found that IL-10 activation of STAT3 in macrophages promotes choroidal neovascular angiogenesis, an important pathological event in blindness, resulting from some forms of age-related macular degeneration. Furthermore, inhibition of the IL-10–STAT3 pathway results in reversal of the phenotype in mouse models of disease. Those previous studies also report that aged pathological macrophages have defective upregulation of *Socs3* in mouse models of disease and consequent permissive IL-10 signaling (Nakamura et al., 2015). We showed that treatment with ADPKD-CM upregulated *SOCS3* appropriately in macrophages in culture (Fig. S3), but we did not examine macrophage expression of this gene in human PKD kidneys. However, because only a small fraction of renal macrophages in ADPKD kidneys were phospho-STAT3 positive, an *in vivo* defect in *SOCS3* expression that promotes permissive IL-10 signaling seems unlikely.

In another system, a recent study has demonstrated a requirement for IL-10 to stimulate macrophage-mediated proliferation in a model of muscle regeneration (Deng et al., 2012). As in wound repair, M2-like macrophages have an important role in muscle growth and regeneration, in part by stimulating the proliferation of myoblasts during the early stage of myogenesis (Cohen and Mosser, 2013). Macrophage-induced proliferation of myoblasts has been shown to require IL-10 stimulation, whereas the proliferation of myoblasts is not altered by IL-10 alone (Deng et al., 2012). As a final example, tumor-associated macrophages, which are programmed by cancer cells to assume phenotypes that support growth, have been shown, in some settings, to acquire a pro-proliferative phenotype in response to IL-10 (Jung et al., 2012b).

Evidence is also presented here that STAT3 is activated in an IL-10-dependent manner during macrophage programming by ADPKD-CM. Small molecule inhibitors of both STAT3 and JAK1, the upstream activator of STAT3, blocked acquisition of the pathological macrophage phenotype. In a recent study, similar STAT3 activation in macrophages has been shown to occur in response to renal tubular-cell-secreted factors, although the effects of this pathway on the functional properties of these cells was not examined (Huen et al., 2015). Here, we have demonstrated colocalization of activated STAT3 and a marker of M2-like macrophages in human ADPKD cystic kidneys, suggesting that the macrophage IL-10–STAT3 pathway is induced during macrophage programming in human disease.

Collectively, these results suggest that the macrophage IL-10–STAT3 pathway could be a good target for therapies to block macrophage-promoted cyst expansion and thus to slow ADPKD disease progression. Relevant to this point, a number of studies have shown that inhibitors of STAT3 activation have efficacy in restraining cyst expansion when administered to mice that model PKD (Leonhard et al., 2011; Takakura et al., 2011). The specific pathways that are dependent on STAT3 activation most relevant to PKD progression have not yet been identified. In those studies, activated STAT3 strongly correlates with the cystic kidney phenotype in both human ADPKD and multiple mouse models of PKD. Activated STAT3 is found within a subset of renal tubule epithelial cells, especially cystic epithelium, as well as in cells within the interstitium, the identities of which have not been determined (Leonhard et al., 2011; Takakura et al., 2011; Talbot et al., 2011). In one of those studies, treatment of a neonatal PKD mouse model with the selective STAT3 inhibitor S31-201 is reported not only to restrain cyst expansion and to reduce the numbers of STAT3-positive nuclei in cyst-lining epithelial cells but also to reduce the numbers of STAT3-positive nuclei in the unidentified interstitial cells (Takakura et al., 2011). Given the demonstration here of activated STAT3 in renal macrophages of ADPKD cystic kidneys (Fig. 6; Fig. S4), we think it likely that the unidentified phospho-STAT3-positive interstitial cells in the cystic kidneys noted in all those previous studies were macrophages. Further, the effectiveness of the STAT3 inhibitors in restraining cyst expansion and PKD progression could be due, at least in part, to inhibitory effects on the STAT3-dependent pro-proliferative functional phenotype of these macrophages. Future studies using conditional inactivation of the STAT3 pathway in PKD-model mice are needed to determine the specific disease-relevant contribution of this pathway.

What are the soluble factors produced by ADPKD cells responsible for programming these macrophages to a pro-proliferative phenotype? In the case of regenerating muscle, where macrophages are stimulated to convert from having an inflammatory phenotype to having an IL-10-producing pro-proliferative phenotype, multiple potential factors have been identified, including prostaglandin derivatives, lipid resolvins, cAMP and adenosine (Cohen and Mosser, 2013). Whether these same components are produced by ADPKD cells or are present in the cystic kidney environment and are capable of inducing macrophage differentiation is yet to be determined.

Following renal I-R injury, specific factors produced by tubule cells are likely to contribute to the development of the distinct M2-like renal macrophage phenotype have been identified. These include M-CSF and GM-CSF, both of which: (1) are produced by tubular epithelial cells; (2) become elevated in the kidney following acute injury; and (3) have been shown to contribute to an M2-like transcriptional profile of macrophages *in vitro* in response to tubule-cell-produced factors (Huen et al., 2015) and *in vivo* following renal injury (Alikhan et al., 2011; Huen et al., 2015; Menke et al., 2009; Zhang et al., 2012). Additionally, after I-R injury, blockade or genetic ablation of either M-CSF or GM-CSF pathways reduce tubular-cell proliferation (Huen et al., 2015; Menke et al., 2009; Zhang et al., 2012). However, the contribution of macrophages to the proliferation and repair that is mediated by these cytokines *in vivo* is only fractional for M-CSF (Menke et al., 2009) and has not been determined for GM-CSF. Also not determined is the potential direct influence of M-CSF or GM-CSF on specific macrophage functions, chiefly the pro-proliferative properties of these cells. We have performed a number of experiments *in vitro* to examine the direct effects of purified GM-CSF and M-CSF, alone or in combination with other cytokines including IL-10, on the pro-proliferative phenotype of macrophages and have detected very little effect on this function (data not shown). However, the potential contribution of these cytokines to macrophage programming by ADPKD-CM has not been definitively determined.

An equally important and unanswered question concerns the identity of the pro-proliferative factors made by macrophages following programming with ADPKD or NHK cell CM. Identification of these factors is likely to provide new targets for the development of effective therapies to slow ADPKD progression. Potential candidates are numerous, and include those factors known to stimulate epithelial cell proliferation and that are produced by macrophages in response to treatment with IL-10 (Jung et al., 2012a), those upregulated in reparative macrophages after renal injury (Huen and Cantley, 2015) and those produced by reparative macrophages during wound healing of any injured tissue (Werner and Grose, 2003). A broad unbiased approach might be most effective in uncovering the identity of these factors.

## MATERIALS AND METHODS

### Antibodies and reagents

ELISA kits for measuring human and mouse IL-10 (#88-7106 and #88-7105, respectively; eBioscience; San Diego, CA) were used according to the manufacturer's directions. The mouse-specific neutralizing anti-IL-10 antibody (#16-7102-85) and a corresponding IgG isotype control antibody (#14-4714-82) were also obtained from eBioscience. For western blots, antibodies specific for phospho-STAT3 (#9145S, diluted 1:500), STAT3 (#9139, diluted 1:1000) and RhoGDI (#2564, diluted 1:1000) were obtained from Cell Signaling Technology (Danvers, MO); the anti-tubulin antibody was from Sigma-Aldrich (#T5201, St. Louis, MO, diluted 1:1000). STAT3 Inhibitor VII (#573103) was obtained from EMD Millipore (Billerica, MA), Stattic (#2798) from Tocris (Fisher Scientific; Pittsburgh, PA) and ruxolitinib (#11609) from Cayman Chemical (Ann Arbor, MI).

### Cell culture

Primary cells obtained from the cavities of cysts on the surfaces of ADPKD kidneys (ADPKD cells) and those obtained from cortical tubule fragments from non-cystic kidneys (NHK cells), human pooled cyst fluids and renal tissues were supplied by the PKD Research Biomaterials and Cellular Models Core at KUMC, the ethical protocols of which comply with federal regulations and were approved by the KUMC Institutional Review Board. The primary cells were cultured up to two passages in ‘ADPKD medium’: Dulbecco's modified Eagle's medium (DMEM)/F-12 (Cellgro 15-090-CV, Mediatech, Manassas, VA) supplemented with 5% FBS, 15 mM HEPES, 5 μg/ml insulin, 5 μg/ml transferrin and 5 ng/ml sodium selenite (ITS, CB40351; Fisher Scientific; Pittsburgh, PA) plus penicillin (100 U/ml) and streptomycin (130 μg/ml) (Pen-Strep). PKD-A cells, an immortalized proximal tubule cell line kindly provided by Robert Bacallao (Indiana University School of Medicine, Indianapolis, IN; Herbert et al., 2013) were maintained in ADPKD medium containing 10% FBS and 5 ng/ml human EGF (E9644; Sigma-Aldrich; St. Louis, MO). THP-1 monocytes were obtained from American Type Culture Collection (ATCC) and maintained in RPMI-1640 medium (R8758, Sigma-Aldrich, St. Louis, MO) containing 10% FBS, 200 μM L-glutamine and Pen-Strep, and were differentiated into macrophages through treatment for 3 days with 200 nM phorbol myristate acetate (PMA; P1585, Sigma-Aldrich) in XVivo-10 (BE04-743Q; Basel, Switzerland) supplemented with 15 mM HEPES. Cells were then washed twice with PBS and further incubated for 3-4 days in XVivo-10 with 15 mM HEPES (Daigneault et al., 2010) before programming. RAW 264.7 cells, obtained from ATCC, were maintained in DMEM (D6429, Sigma-Aldrich) medium containing 10% FBS, 200 µM L-glutamine and Pen-Strep. All cell lines were tested for mycoplasma contamination using a kit (#LT07-118; Lonza) according to the manufacturer's directions.

### Isolation of BMDMs

Primary BMDMs were isolated from 6- to 8-week-old wild-type Balb/cJ or IL-10-deficient mice (Balb/cJ background), obtained from JAX labs (Stock No. 004333) using similar methods to that described previously by Zhang et al. (2008). Briefly, cells were flushed from mouse tibias and femurs with sterile PBS, washed and centrifuged (1200 ***g***, 5 min) and cultured (5.0×10^6^ cells) on non-tissue-culture plastic 100-mm Petri dishes in 10 ml of macrophage complete medium (DMEM containing 10% heat-inactivated FBS, 2 mM L-glutamine, Pen-Strep and 10% L929 fibroblast-cell-conditioned medium). Cells were incubated at 37°C, under 5% CO_2_ for 6 days with the addition of 4 ml fresh macrophage complete medium after 3 days. Non-adherent cells were then removed, and the adherent cells (macrophages) were placed into 5 ml of cold PBS for 30 min at 4°C. Cells were then scraped from the dish, centrifuged at 1200 ***g*** for 5 min and resuspended in basal medium (DMEM containing 2 mM glutamine, Pen-Strep) in preparation for Transwell-insert proliferation assays, or in DMEM containing 5% heat-inactivated FBS, 2 mM L-glutamine and Pen-Strep for plating and programming with ADPKD-CM or NHK-CM.

### Production of ADPKD-CM for programming of macrophages

Primary ADPKD cyst cells were seeded (3.7×10^6^) in a 15-cm plate and grown overnight in ADPKD medium. Cells were washed twice in PBS, and medium was switched to 20 ml XVivo-10 supplemented with 15 mM HEPES, and were incubated for 3 days. Conditioned media were collected, centrifuged at 600 ***g*** and stored at −80°C.

### Programming of macrophages with ADPKD cyst cells or ADPKD-CM

THP-1 macrophages (3.5×10^6^/well in a 6-well tissue-culture plate for incubation with ADPKD cells; 2×10^6^/well for incubation with ADPKD-CM) were incubated with ADPKD cyst cells (1×10^4^/well in a volume of 2 ml) in XVivo-10 supplemented with 15 mM HEPES medium for 3 days, or were incubated with ADPKD-CM (2 ml) for 3 days. Media were then replaced with basal medium (DMEM containing 2 mM glutamine, Pen-Strep) for 1 day, and this conditioned medium was collected and clarified by centrifugation at 600 ***g***. FBS (0.1%) was added to each CM before incubation with cyst cells to assess proliferative activity.

### Programming of RAW 264.7 cells with ADPKD-CM

RAW cells were seeded at 1×10^6^/well in a 6-well tissue-culture plate in DMEM containing 10% FBS, 200 µM L-glutamine and Pen-Strep. After 24 h, cells were washed with PBS and incubated for 18-24 h with ADPKD-CM (medium was XVivo-10 with 15 mM HEPES). Media were then replaced with basal media for 1 day, and this conditioned media (CM) was collected and clarified by centrifugation at 600 ***g***.

### Proliferation assays of ADPKD cells

#### Direct co-culture proliferation assays

ADPKD cells were plated in triplicate at a density of 5×10^4^ cells/well in a 6-well tissue-culture plate. After 24 h of incubation, media were changed for an ATP-depleting starvation medium [glucose-free DMEM (11966, Life Technologies) containing 2% FBS, 10 mM deoxyglucose and Pen-Strep] and cells were re-incubated for 24 h before the removal of medium and the addition of RAW cells (5×10^4^) in basal medium containing 0.1% FBS. After 3 days of further incubation, cells were collected and fixed in 2% PFA in PBS. The number of ADPKD cells in each sample was determined by counting under a microscope using a hemocytometer. ADPKD and RAW cells were easily distinguished by size and morphology.

#### Transwell-insert proliferation assays with BMDMs

ADPKD cells were plated in triplicate at a density of 2.0×10^5^ cells per well in a 6-well plate and incubated for 24 h before the replacement of media with ATP-depleting media and a further 24 h of incubation. Media were then changed into basal media containing 0.1% serum before the insertion of the Transwell filters (0.4 μm) onto which BMDMs (5.0×10^5^ in 1 ml of basal medium with 0.1% FBS) were added. Cultures were incubated for 3 days, after which the Transwell insert was removed, and the ADPKD cells collected and fixed in 2% PFA in PBS. Quantitative microscopic counting was performed manually to determine the number of ADPKD cells.

#### Proliferation assays in response to macrophage-conditioned media

Cyst epithelial cells (PKD-A or primary ADPKD cells) were seeded in triplicate in a 24-well tissue-culture plate (1.2×10^4^ cells/well) in ADPKD medium and incubated at 37°C for 24 h before changing media to the ATP-depleting starvation medium. Following an additional 24 h of incubation at 37°C, media were replaced with either basal media containing 0.1% or 10% FBS, or with CM collected from programmed or unprogrammed macrophages (in basal media) to which FBS (0.1%) had been added, and cells were incubated for 3 days. Cells were then frozen at −80°C prior to lysis in buffer containing CyQUANT^®^ GR dye (Life Technologies), according to manufacturer's directions. Quantitative measurement of fluorescence was performed using a Synergy™ 2 microplate reader (BioTEK Instruments, Inc., Winooski, VT).

### Immunoblots, immunohistochemistry and immunofluorescence

Immunoblot bands were detected using the Odyssey system from Li-Cor Biotechnology (Lincoln, NE). The IRB-approved PKD Biomaterials Research Core laboratory at KUMC supplied all human kidney tissue. The research used only de-identified archival materials derived from procedures already performed in the course of the patient's clinical care. Informed consent was obtained from all patients prior to surgery and tissue donation. Sections (4 µm) from formalin-fixed paraffin-embedded kidney tissues (human) were de-paraffinized and steamed in 0.01 M citrate buffer (pH 6.0) for 20 min (steamer #HS900, Black & Decker, Madison, WI). For immunohistochemistry, sections were then incubated in 3% H_2_O_2_ followed by serum from the host animal in which the relevant secondary antibody was generated. Samples were then incubated with either anti-CD163 (MS-1103, Thermo Scientific, Rochester, NY) or anti-phospho-STAT3 antibodies overnight at 4°C. Appropriate secondary antibodies (ImmPRESS, Vector Laboratories, Burlingame, CA) were then applied for 30 min at room temperature, followed by incubation with DAB substrate (Vector Laboratories) and hematoxylin counterstain, prior to visualization with light microscopy. For immunofluorescence, de-paraffinized steamed sections were incubated in 2.5% horse serum in PBS for 30 min, followed by co-incubation with anti-CD163 and anti-phospho-STAT3 antibodies overnight at 4°C. Secondary antibodies (Cy™3-conjugated anti-rabbit, #711-165-152; and FITC-conjugated anti-mouse, #715-095-150; Jackson ImmunoResearch; West Grove, PA) were then applied. Samples were mounted in Prolong^®^ Gold Antifade with DAPI (#P-36931; ThermoFisher Scientific) and visualized by using fluorescence microscopy. Red, green and blue images were merged using Photoshop CS6. For quantification, cells were counted in 5-7 high-powered fields per kidney section. Field images were taken with the 40× objective.

### qRT-PCR

Using a mortar and pestle, renal tissues, stored at −80°C, were ground to a fine powder under liquid N_2_ and lysed immediately by the addition of RLT lysis buffer from the RNeasy Miniprep kit (Qiagen, Valencia, CA). Samples were further homogenized using QIAshredder columns (Qiagen, Valencia, CA). Total RNA from these lysates, as well as from cell samples, was purified according to the RNeasy Miniprep kit directions and then analyzed by the KUMC Genome Sequencing Facility for quality, determined with an Agilent 2100 Bioanalyzer. RNAs with an RNA integrity number (RIN) of at least 8 were used for qRT-PCR analysis. cDNA was synthesized using a kit (#1725037) from Bio-Rad (Hercules, CA), and qRT-PCR analysis was performed on a Bio-Rad CFX96 real-time PCR machine using Sybr Green mix (#1725271) from Bio-Rad. Expression of human genes was normalized to that of human *OAZ1.* The following primers were used: human *IL10*, 5′-TCAAGGCGCATGTGAACTCC-3′ and 5′-GATGTCAAACTCACCAGGCT-3′; human *SOCS3*, 5′-GGCTCAGCCCCAAGGAC-3′ and 5′-GAGCCAGCGTGGATCTG-3′; and human *OAZ1*, 5′-CACCATGCCGCTCCTAAG-3′ and 5′-GAGGGAGACCCTGGAACTCT-3′. The efficiency of each primer pair was determined using the DART-PCR program (Peirson et al., 2003). Using the gene-specific efficiencies, the mRNA relative abundance was calculated according to the method described previously by Pfaffl (2001), and normalized to the mean of human *OAZ1* expression.

### Statistical analysis

Data between two groups were compared using the two-tailed *t*-test calculated with Prism (v4.0, GraphPad, La Jolla, CA). *P*-values<0.05 were considered significant, as indicated in the figures. Data are presented as mean±s.e.m.
